# A case report of an oral hemangioma with unusual features

**DOI:** 10.1002/ccr3.9479

**Published:** 2024-11-06

**Authors:** Samaneh Salari, Nooshin Mohtasham, Mahrokh Imanimoghaddam, Maryam Amirchaghmaghi, Mahsa Fayyazi

**Affiliations:** ^1^ Oral and Maxillofacial Medicine, Oral and Maxillofacial Diseases Research Center Mashhad University of Medical Sciences Mashhad Iran; ^2^ Oral and Maxillofacial Pathology, Oral and Maxillofacial Diseases Research Center Mashhad University of Medical Sciences Mashhad Iran; ^3^ Oral and Maxillofacial Radiology, Oral and Maxillofacial Diseases Research Center Mashhad University of Medical Sciences Mashhad Iran; ^4^ Oral and Maxillofacial Medicine Mashhad University of Medical Sciences Mashhad Iran

**Keywords:** capillary hemangioma, hemangioma, oral cavity, vascular malformation

## Abstract

**Key Clinical Message:**

A 26‐year‐old patient with swelling on the lingual surface of the mandible in the incisors area was referred to the Faculty of Dentistry at Mashhad University of Medical Sciences. After conducting clinical, radiographic, and pathological examinations, the patient was diagnosed with capillary hemangioma. This study explores the clinical manifestation and unusual behavior of intraoral hemangioma.

**Abstract:**

Hemangioma is a tumor that occurs due to the proliferation of endothelial cells in 4%–5% of infants and affects the head and neck region in almost 70% of cases. The tumor in the mouth can appear as a soft, smooth, or lobulated mass with or without a base and varying in size from a few millimeters to a few centimeters. This article reports a case of hemangioma with unusual clinical features in a 26‐year‐old male patient.

## 1 INTRODUCTION

A hamartoma is a benign tumor of normal cells in an abnormal location. However, hamartomas maintain growth cessation without the potential for further growth or malignant transformation. Hamartomas are commonly seen in the lungs, kidney, liver, and spleen and rarely occur in the orofacial region. Various hamartomatous lesions including hemangiomas, lymphangiomas, exostosis, dens invaginatus, dens evaginatus, odontomas, nevi, and cherubism.^1^

Hemangiomas are benign soft tissue vascular tumors and true neoplasms of endothelial cells that are often present at birth or appear several weeks after birth and grow and spread rapidly as the child grows.

Clinically, hemangiomas may occur in two forms: infantile or congenital hemangiomas. Infantile hemangioma develops in the first 2 months of life and grows rapidly until 6 months of age and followed by a period of regression, but congenital hemangioma which is fully developed at birth and occurs in two varieties, rapidly involuting congenital angioma (RICH) and noninvoluting congenital hemangioma (NICH). RICH shows early regression, with full involution by 9–14 months of age, and NICH grows proportionately with the child.^1^, ^2^

Head and neck hemangiomas are histologically divided into three types, namely capillary, cavernous, and mixed. Capillary hemangiomas are more common than other types.^3^ This benign tumor is significantly more common in women than in men (3:1) and occurs in 80% of cases.^4^ Intraoral capillary hemangiomas are very rare and account for 0.5%–1.0% of all intraoral neoplasms.^5^, ^6^ The most common site of occurrence of these benign tumors is the oral cavity, lips, tongue, and buccal mucosa.^3^ The clinical manifestation of oral cavity lesions is diverse and different layers including skin and even muscles are involved, although the adjacent bone is rarely affected.^7^ The lesion does not pose any risk to the patient and surgical treatment is only necessary if the aesthetic manifestation is affected, chewing is impaired or bleeding occurs.

## 2 CASE PRESENTATION

### 2.1 Case History/Examination

**FIGURE 1:**
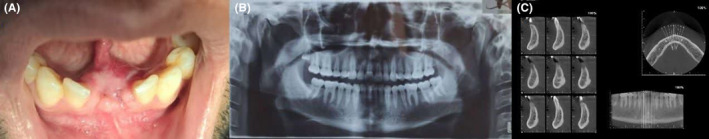
Clinical figure (A). Panoramic radiograph (B). CBCT (C).

A 26‐year‐old male patient with complaints of swelling in the lingual area of the lower anterior teeth was referred to the Faculty of Dentistry, Department of Oral Medicine, Mashhad University of Medical Sciences, Iran. No history of systemic disease or drug use was mentioned. In addition, no lymphadenopathy or facial asymmetry was observed on extra‐oral examination. On intraoral examination, a prominent pinkish nodular lesion with a rubbery consistency and telangiectasia on the surface of the lesion was observed. The lesion was approximately 2.5 cm in size. On the lingual surface of the mandible in the anterior portion of the lower jaw in the missing mandibular anterior teeth area. The patient had the course of the lesion for approximately 10 months, sometimes with trauma‐related bleeding. Approximately 1 month previously, mandibular central incisors were extracted due to tooth mobility, and the area was curetted. However, the lesion had recurred in the same location several days after the extraction (Figure 1).

In the patient's dental records, the previous radiographs (OPG) were reviewed, and radiolucency with clear boundaries was observed in the area of mandibular central incisors. Based on the clinical manifestation, location, and history of tooth mobility and the radiolucency observed on the OPG, additional CBCT radiography was requested to rule out central lesions, including variation in the position of the mental fossa and residual cysts. However, no pathological findings were reported in the area of the mandibular anterior teeth.

## 3 METHODS

**FIGURE 2:**
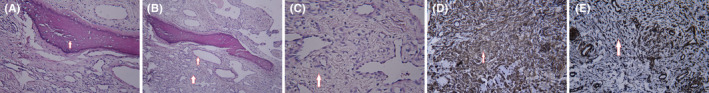
Pathology with H&E staining, overview of the proliferation of endothelial cells, blood vessels, and bone tissue (magnification 200× and 400×) (A–C). Immunohistochemical staining (cd68) of background histiocyte cells was positive (D). Immunohistochemical staining (vimentin) was positive in connective tissue cells (E).

To complete the clinical examination, aspiration of the lesion was performed, and passive blood was observed. Based on the results of clinical examinations and the absence of intraosseous lesions in the area of the mandibular anterior teeth, the lesion was clinically diagnosed as a vascular malformation or a hemangioma. To confirm this, the non‐invasive ultrasound technique was also used. The results showed that the lesion was a mass with unclear boundaries and heterogeneous echo containing three calcified areas with dimensions of 24, 13, and 16 mm in the lingual area of the gingiva. The mandibular anterior region had a significant blood supply on the Doppler scan and ultrasound; the lesion was diagnosed as potentially calcified hemangioma, and oral choristoma. Choristoma was proposed due to the few vessels and irregular calcification. According to the clinical diagnosis of vascular lesions, a sample was collected from the lesion. During histopathological examination, the proliferation of endothelial spindle cells was observed with the formation of numerous blood vessels, often with capillary vessels that divided into parts by the fibrous connective tissue. Some bone trabeculae were also observed in the lesion, and the diagnosis of calcified capillary hemangioma and choristoma was made (Figure 2). According to spindle cells, histiocytes, and bone cells in histopathology, immunohistochemical examination was recommended to rule out relevant lesions.

**FIGURE 3:**
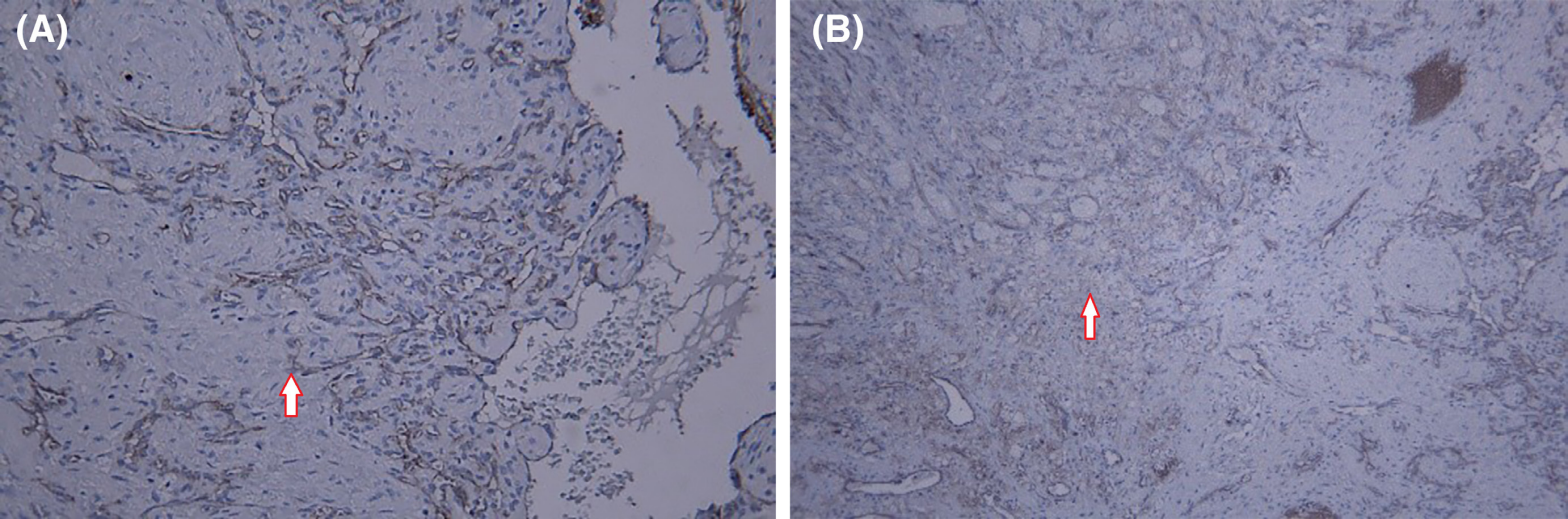
Immunohistochemical staining (CD31) related to positive endothelial cells (magnification ×100 and ×200) (A, B).

1. Immunohistochemical examination of vimentin was performed on connective tissue lesions observed in the background and reported as positive (Figure 2).
2. S100, Sox10, inhibin, and calretinin were performed to detect and rule out negative nerve lesions, paragangliomas, and neural crest lesions.
3. CD68 was performed to detect lesions originating from histiocytes, such as histiocytomas, which were negative and ruled out.
4. SMA was performed to examine the smooth muscle of the vessel wall with the origin of pericytes such as hemangiomas and pericytomas, and it was negative.
5. CD31 was performed to examine the lesions originating from endothelial cells. This was positive and suggested the vascular origin of the lesion. Therefore, due to the negativity of other markers, the diagnosis of capillary hemangioma was confirmed (Figure 3). Based on the patient choice for the complete removal of the lesion, the patient was referred to a maxillofacial surgeon, and after CT angiography of the lesion, a complete operation of the lesion was performed.

## 4 CONCLUSION AND RESULTS

Capillary hemangioma can be diagnosed differentially from other lesions such as vascular malformations or inflammatory hyperplasia, and histopathological findings are very helpful in the final diagnosis. Because hemangioma lesions are asymptomatic, the location and course of the lesion must be considered in the diagnosis. Vascular hemangioma lesions with unusual manifestations and locations are often regarded as a diagnostic problem for general dentists. When removing the hemangioma through a surgical incision, severe bleeding may occur, although in our case this did not occur during tooth extraction and surgery. Due to the small size of the lesion, easy access, and low risk of bleeding, surgical treatment was chosen in our case. However, due to the unavailability of the patient, further follow‐up was not possible.

## 5 CASE DISCUSSION

Hemangiomas are benign tumors of the capillary endothelium that occur in newborns between two and 8 weeks of age, grow rapidly in the first year, and resolve in 50% of cases by the age of 5 years.^8^ Hemangioma is one of the most common soft tissue tumors in the head and neck region. However, it rarely occurs in the oral cavity and is congenital in most cases, while its occurrence in adults is very rare.^9^ On the other hand, hemangioma is the most common tumor in infants, occurring in 5%–10% in the first year of life. It occurs as a single lesion in 80% of cases and as a multiple lesion in the rest. The clinical manifestation can vary depending on the surface or depth of single or multiple lesions.^9^ It can bleed spontaneously or due to trauma and is often painless. In our case, it was a pink, baseless mass with a smooth surface and with no symptoms.

Hemangiomas affect various areas of the oral cavity, including the tongue, lips, and buccal mucosa, as well as the mandible. When the lesions occur in the gingiva, the interdental papilla is the most common site of infection.^10^ Matsumoto et al. studied 31 patients with intraoral hemangioma and found that most lesions were located on the oral mucosa (45.2%), followed by the tongue (35.5%), lip (9.7%), gingiva (6.5%), and palate (3.2%).^5^ In the case of our patient, the lesion occurred in the lingual part of the anterior gingiva of the mandible. After initially ruling out bone involvement, the lesion was differentially diagnosed as inflammatory hyperplasia. However, taking into account the distance from the marginal gingiva, the observed manifestation, and the blood discharge during aspiration, inflammatory hyperplasia was ruled out, and vascular lesions were ultimately suggested as the most likely diagnosis, although the location was unusual for intraoral hemangiomas.

The differential diagnoses of intraoral capillary hemangioma are vascular malformations and pyogenic granuloma, which are histologically similar and can be differentiated by biopsy and immunohistochemical examination.^11^ Hemangiomas are true benign neoplasms of endothelial cells that may be present at birth but vascular malformations are localized defects of vascular architecture with abnormal torturous and enlarged vascular channels always present at birth. Over time, hemangiomas become smaller (involute) and lighter in color and may leave a scar, but vascular malformations do not involute spontaneously and may become more apparent as the child grows.^1^

Since the history and course of the lesion are very helpful in diagnosing vascular anomalies, the unclear history of these lesions may lead to incorrect diagnoses and inappropriate treatments. In our case, such a misdiagnosis was made by a general dentist. To confirm the diagnosis of vascular lesions, histopathological examination is considered the diagnostic standard. Radiographic examination is necessary to differentiate such lesions from other intraosseous lesions or central hemangiomas.^12^

On the initial panoramic radiograph, radiolucency was observed in the mental fossa region; therefore, due to the inaccuracy of the OPG radiograph in the anterior mandible, an additional CBCT imaging was required for a detailed examination of the area. According to the CBCT imaging, lesions of bony origin and intraosseous hemangiomas could be excluded. Because it was not observed in the CBCt, it is possible that the radiolucency observed in the panoramic was related to the mental fossa. In addition, the patient had a history of loose teeth that appeared unrelated to the lesion and was likely caused by occlusal trauma.

Calcified spots were unexpectedly observed on ultrasound of the lesion, which was differentially diagnosed as choristoma.^13^ Choristomas are rare benign lesions that result from the growth of normal tissue in an abnormal location, and their etiology is unknown.^14^ Considering that choristoma in the lingual gingiva is very rare and that the clinical, radiographical, and histopathological evidences confirmed the presence of a vascular lesion. The calcifications were probably due to the slowing of peripheral blood flow and the consequent thrombosis and mineralization within the vessels.^15^ The rare thrombosis and phlebitis in hemangioma in our case were confirmed by the ultrasound and histopathological examination of the lesion. Hemangioma treatment is only necessary when there is a functional disorder such as malaise and recurrent bleeding or for aesthetic reasons.^16^ Treatment includes surgery, laser, sclerosing agents, embolization, and corticosteroid therapy.^17^ Treatment of oral hemangioma depends on various factors such as age, size, and extent of the lesion, site of involvement, and clinical features.^17^

Given the unusual location of the lesion on the gum, this case is of particular importance as dentists may mistakenly consider it a periapical lesion or a hyperplasia lesion.

## AUTHOR CONTRIBUTIONS

**Samaneh Salari:** Writing – original draft. **Nooshin Mohtasham:** Data curation. **Mahrokh Imanimoghaddam:** Resources. **Maryam Amirchaghmaghi:** Writing – review and editing. **Mahsa Fayyazi:** Writing – original draft; writing – review and editing.

## FUNDING INFORMATION

This research did not receive any specific grant from funding agencies in the public, commercial, or not‐for‐profit sectors.

## CONFLICT OF INTEREST STATEMENT

The authors report no conflict of interest.

## CONSENT

Written informed consent was obtained from the patient to publish this report in accordance with the journal's patient consent policy.

## Data Availability

Data can be obtained from the corresponding author upon request.
